# Advances in Noninvasive Biomarkers for Nonalcoholic Fatty Liver Disease

**DOI:** 10.3390/metabo13111115

**Published:** 2023-10-29

**Authors:** Georgiana-Emmanuela Gîlcă-Blanariu, Daniela Simona Budur, Dana Elena Mitrică, Elena Gologan, Oana Timofte, Gheorghe Gh Bălan, Vasile Andrei Olteanu, Gabriela Ștefănescu

**Affiliations:** 1 Gastroenterology Department, “Grigore T. Popa” University of Medicine and Pharmacy, 700115 Iași, Romania; georgiana-emmanuela.gilca@umfiasi.ro (G.-E.G.-B.); dana.mitrica@umfiasi.ro (D.E.M.); elena.gologan@umfiasi.ro (E.G.); oana.timofte@umfiasi.ro (O.T.); gheorghe-g-balan@umfiasi.ro (G.G.B.); vasile-andrei.olteanu@umfiasi.ro (V.A.O.); 2 Department of Gastroenterology, “Sf Spiridon” County Clinical Emergency Hospital, 100115 Iași, Romania

**Keywords:** biomarkers, nonalcoholic fatty liver disease, nonalcoholic steatohepatitis, markers, serological markers, fibrosis

## Abstract

Nonalcoholic fatty liver disease (NAFLD) currently represents one of the most common liver diseases worldwide. Early diagnosis and disease staging is crucial, since it is mainly asymptomatic, but can progress to nonalcoholic steatohepatitis (NASH) or cirrhosis or even lead to the development of hepatocellular carcinoma. Over time, efforts have been put into developing noninvasive diagnostic and staging methods in order to replace the use of a liver biopsy. The noninvasive methods used include imaging techniques that measure liver stiffness and biological markers, with a focus on serum biomarkers. Due to the impressive complexity of the NAFLD’s pathophysiology, biomarkers are able to assay different processes involved, such as apoptosis, fibrogenesis, and inflammation, or even address the genetic background and “omics” technologies. This article reviews not only the currently validated noninvasive methods to investigate NAFLD but also the promising results regarding recently discovered biomarkers, including biomarker panels and the combination of the currently validated evaluation methods and serum markers.

## 1. Introduction

Nonalcoholic fatty liver disease (NAFLD) represents a spectrum of diseases associated with metabolic disorders, which include entities from simple steatosis to nonalcoholic steatohepatitis (NASH), with potential progression to liver cirrhosis and its complications. There has been a notable increase in the global burden of obesity and associated metabolic pathologies over the past years, including an increase in the prevalence of nonalcoholic fatty liver disease (NAFLD), reaching an estimated global prevalence of 30% [1].

Considering the ongoing obesity epidemic, beginning in the early stages of life, and the increase in various metabolic factors, the prevalence of NAFLD along with the proportion of those with advanced liver disease is projected to continue to increase [2]. Indirect estimations of the prevalence of NASH among NAFLD patients have reported varying results, ranging from around 6% among NAFLD patients who underwent a random liver biopsy to 59% among NAFLD patients with a clinical indication for a liver biopsy [3], with an overall average prevalence of NASH estimated to be between 1.5% and 6.45% [4].

NASH is characterized by various pathophysiological changes in the liver, beginning with hepatocyte stress, injury, apoptosis, and inflammation, leading to fibrosis [5]. In the absence of early diagnosis and adequate management, NASH may progress to cirrhosis, with a prevalence of severe F3 and F4 fibrosis related to NASH rapidly increasing in the US and globally [6]. There is an associated risk of liver-related morbidity and mortality, which rises as the degree of liver fibrosis progresses [7]. NAFLD and NASH also negatively impact the health-related quality of life, even in the precirrhotic stages [8], supporting the demand for early diagnosis and effective treatments. Currently, a NASH diagnosis relies on the use of a liver biopsy, with several criteria to be fulfilled for diagnosis, including macrovesicular fatty changes of hepatocytes, ballooning degeneration of hepatocytes, and infiltration of inflammatory cells [9]. Identification of NAFLD patients, and especially the subgroup who progress to NASH among NAFLD patients, is essential in order to optimally manage these cases, and various noninvasive tools have been developed in this regard, although no unique biomarker is solely acknowledged to meet the requirements sufficiently. The aim of this article is to review the currently validated biomarkers and scores and to highlight the promising results regarding potential biomarkers and biomarker panels that are useful in the evaluation of NAFLD and NASH.

## 2. Currently Used Noninvasive Methods for NAFLD Evaluation

Since NAFLD is related to metabolic syndrome, it can be initially managed by changing the lifestyle, but very few people can sustain this long-term, which is the reason why as the condition progresses, physicians involve pharmacological therapy in patient management [10]. Therefore, the necessity of accurately establishing the stage of steatosis or fibrosis is vital in correctly placing the diagnosis in a therapeutic context. At the moment, the diagnosis is assessed by starting with the patient history, physical examination, and laboratory tests. Medical comorbidities, such as obesity, diabetes, discovering features of advanced liver disease such as a firm liver or dyslipidemia, and abnormalities in the liver biochemistry like elevated aminotransferases, all represent arguments for screening for NAFLD [11]. Abdominal ultrasonography is also a common modality to approximate the liver steatosis grade in a patient with an incidental elevation of transaminases [12]. The Fibrosis-4 Index for Liver Fibrosis (FIB-4) is a score that is very helpful in detecting patients with a higher risk of developing advanced fibrosis, and this is why it is used as a first step in ruling out this stage of disease (it has a negative predictive value of 90–95%). It is a very simple index that is based on age, platelet count, alanin transaminase (ALT), and aspartate transaminase (AST) [13]. A statistically better test for progressive fibrosis is the Enhanced Liver Fibrosis Panel (ELF) or imaging-based elastography methods such as vibration-controlled transient elastography (VCTE), shear-wave elastography (SWE), acoustic radiation force impulse (ARFI) imaging, and magnetic resonance elastography (MRE). Increased liver stiffness can be a consequence of various processes, such as passive congestion or inflammation, or it can be due to infiltrative disease, but the most important is fibrosis [11].

VCTE or Fibroscan^®^ represents the first elastographic method that has been FDA-approved, and it is useful not only for assessing fibrosis but also for evaluating the steatosis grade using a controlled attenuation parameter (CAP). SWE measures liver stiffness with the help of ultrasonography as well as ARFI elastography. MRE is performed during an MRI scan and similar to VCTE, it is able to assess steatosis with the assistance of an additional parameter, the proton density fat fraction (PDFF) [14]. In a study conducted by Cassinotto et al., the AUROC values for Supersonic Shear Imaging, FibroScan, and ARFI for detecting advanced fibrosis F4 were 0.88, 0.87, and 0.84, respectively, leading to the conclusion that the SWE prediction value is superior compared with VCTE and ARFI [15]. They also highlighted some factors that interfere with the accuracy of the liver stiffness methods such as obesity, the presence of metabolic syndrome, or diabetes.

There are a plethora of currently available validated algorithms proposed for diagnosing and staging fibrosis such as the FibroTest, FibroMeter, Hepascore, NAFLD fibrosis score, BARD score, AST/platelet ratio index (APRI), or Hepamet fibrosis score, which are systematically presented in Table 1.

## 3. Emerging Biomarkers

**Figure 1:**
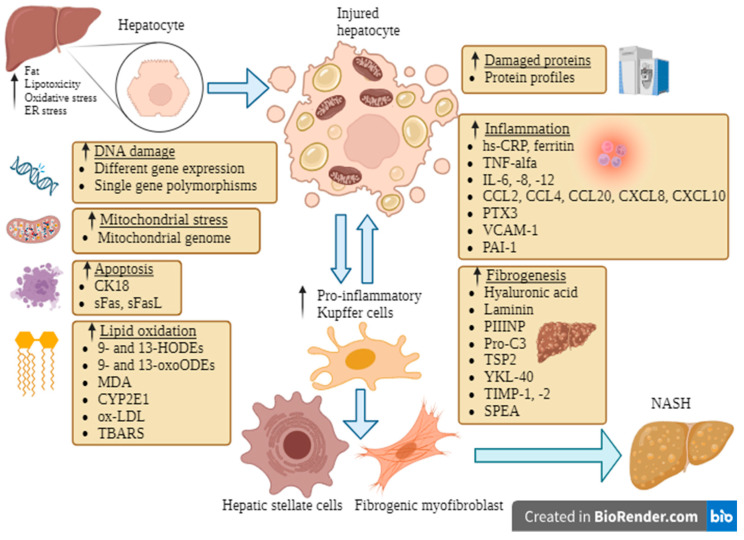
An overview of the mechanisms involved in NAFLD and progression to NASH, pointing to the potential biomarkers.

The pathophysiology of NAFLD and NASH is extremely complex and includes several processes. For each of these processes, several markers can be identified, contributing to a large pool of biomarkers that are potentially useful in assessing disease severity and progression (Figure 1).

### 3.1. Biomarkers of Fibrogenesis

Fibrosis is one of the pathophysiological processes involved in advanced liver disease and represents the excessive development of fibrous connective tissue in the liver, reflecting the progression of NAFLD. During fibrogenesis and fibrinolysis, fragments of the extracellular matrix (ECM) are released into the blood circulation, and measuring the serum level of these molecules could evaluate the degree of liver fibrosis [19]. 

**Hyaluronic acid (HA)** is one of the most studied potential biomarkers for fibrosis in NAFLD. In a clinical study including 79 patients with biopsy-proven NAFLD, Suzuki et al. identified a significant correlation between the HA level and the degree of liver fibrosis. This finding suggests that HA is only useful for detecting advanced stages of fibrosis (AUROC = 0.89), and it is not relevant for a mild degree of fibrosis (AUROC = 0.67 for any levels of fibrosis) [20]. Malik et al. also concluded that HA obtained a good AUROC (0.77) only for advanced fibrosis [21]. Lydatakis et al. have determined the serum levels for HA and laminin in a group of 50 patients with NASH, of which 23 were diagnosed with fibrosis and 27 were without fibrosis [22]. For HA, the conclusion was consistent with the previous research and for laminin, its inability to stage fibrosis was identified. Sakugawa et al. also confirmed, with promising results, the usefulness of HA in diagnosing and staging NASH and fibrosis [23]. Moreover, they spotlighted the fact that the association of HA and type VI collagen 7S domain has a high negative predictive value (95.2%). According to another study, HA obtained the best AUROC (0.885) for independently predicting severe fibrosis [24]. 

**Laminin** represents another direct marker of matrix deposition, and it is an abundant noncollagenous glycoprotein [10]. In the studies performed by V.N. dos Santos et al. [25] and Ratziu et al. [26], respectively, significant values for AUROC, sensitivity, specificity, PPV, and NPV were achieved. Regarding **procollagen III amino-terminal peptide (PIIINP)**, there are conflicting results. This peptide is released during the synthesis and deposition of type III collagen, and Monarca et al. acknowledged that it is not a good discriminator between NAFLD and ALD (alcohol-related liver disease) [17,27]. However, in 2013, a study [28] validated its ability to detect and assess NASH (AUROC of 0.77–0.82 in patients with F0-2 fibrosis and 0.82–0.84 in patients with F0-3 fibrosis).

**Pro-C3** is the pro-peptide of type III collagen and a neo-epitope-specific competitive ELISA for PIIINP [10]. According to Nielsen et al., it is specific for fibrogenesis rather than degradation [29]. This molecule is correlated to the percentage of fibrosis and fat (*p* = 0.01–0.0007) [30]. Daniels et al. not only confirmed that Pro-C3 increased with the fibrosis stage (obtaining an AUROC of 0.81 for identifying patients with F3) but also developed a Pro-C3-based score called ADAPT that accurately identified patients with NAFLD [31]. The **ADAPT score** consists of combining age, diabetes status, platelet count, and Pro-C3, and it has an AUROC higher than Pro-C3 alone (0.86). A relatively recent study suggested that bariatric surgery could contribute to the decrease in the levels of Pro-C3 as a result of improvements in metabolic and liver parameters [32]. A meta-analysis concluded that Pro-C3 or a panel incorporating Pro-C3 has comparable diagnostic accuracy with the currently validated ELF test or FIB-4, which are recommended by guidelines [33].

**Chondrex (YKL-40)** is another type of protein involved in extracellular matrix remodeling, and it is a feasible biomarker of liver fibrosis in NAFLD patients according to Kumagai et al. [34]. **Thrombospondin 2 (TSP2)** has various functions including collagen formation, and Kimura et al. analyzed the relation between NAFLD and serum TSP2 levels, resulting in an AUROC of 0.82 for predicting severe fibrosis [35], while in the evaluation conducted by Kozumi et al., an AUROC of 0.856 for predicting advanced fibrosis was obtained [36].

As far as markers for fibrolysis are concerned, the **tissue inhibitors of metalloproteinases 1 and 2 (TIMP-1, TIMP-2)** are potentially useful markers for assessing fibrosis. Abdelaziz et al. obtained promising values for sensitivity and specificity for both enzymes and an AUROC of 0.971 for TIMP-1 and 0.951 for TIMP-2 [37]. However, there is a study [38] that revealed that there was no significant correlation between the serum level of TIMP-1 and the histological aspect. Measuring **serum prolidase enzyme activity (SPEA)**, NASH can be differentiated from simple steatosis with an AUC of 0.85. This enzyme, which is involved in collagen breakdown, is correlated with the liver collagen content in hepatic fibrosis [39,40]. The **Enhanced Liver Fibrosis Panel (ELF)** is a blood test consisting of three markers of matrix turnover: hyaluronic acid, tissue inhibitors of metalloproteinase 1 (TIMP-1), and aminoterminal peptide of pro-collagen 3 (PIIINP), and it performs well in detecting advanced fibrosis, with an AUROC of 0.90 [13].

Besides evaluating markers of matrix deposition and degradation, fibrosis can be assessed through cytokines and chemokines such as the platelet-derived growth factor (PDGF), transforming growth factor (TGF)-alpha, and (TGF)-beta [41,42]. Regarding **endothelin-1** (ET-1), Degertekin et al. found a correlation with the stage of fibrosis and also a significant relation among insulin resistance, ET-1 levels, and the grade of hepatic fibrosis [43]. The **PRTA score** combines the platelet-derived growth factor receptor beta (sPDGFRβ) levels with platelet counts and albumin and has a great diagnostic value for advanced hepatic fibrosis, with an AUROC superior to Fib-4, APRI, and AST/ALT [44].

### 3.2. Biomarkers of Inflammation

The progression of NAFLD into NASH involves inflammatory mechanisms, which can be evaluated through inflammatory markers and mediators such as high-sensitivity C-reactive protein (hs-CRP), ferritin, tumor necrosis factor (TNF), interleukins, and others. Even if some of these are not specific for liver inflammation, they are still useful for evaluating the inflammatory metabolic state and the progression of the liver injury. The studies on **hs-CRP** are conflicting [45], since there are results that show a correlation between the serum level of hs-CRP and the presence of steatohepatitis and severe fibrosis [46,47,48], while other studies highlight a weak relationship between the levels of hs-CRP and the degrees of steatosis [49] or even no value of hs-CRP in discriminating between simple steatosis and steatohepatitis [50]. It has been suggested that the accumulation of fat—both in the adipose tissue and in liver steatosis—is leading to the high levels of CRP in obese patients [51]. Another biomarker currently available to evaluate inflammation and readily available in clinical practice is **ferritin**, and its role in NAFLD has also been studied, with some studies suggesting [52] that a serum level higher than 1.5 X the upper limit of normal is associated with more severe histologic features and predicts the development to advanced fibrosis [13].

Insulin resistance underlies the development of NASH, and **TNF-alfa** plays an important role in this condition as an inflammatory mediator [53]. A cross-sectional study from China [54] demonstrated that TNF-alfa levels are associated with the development of NAFLD and NASH. A few more studies are showing the correlation between the tumor necrosis factor and the presence and severity of NASH [55,56,57]. However, in combination with the assessment of IL-8 and pyroglutamate, the obtained values were more significant [58,59]. Interesting results were acquired by measuring mRNA levels for TNF-alfa, where a lower value for microRNA 144 (miR-144) seemed to be responsible for promoting TNF-alfa induction [60].

Another proinflammatory cytokine is **IL-6**, for which there are indicative studies that suggest its ability to detect inflammation-related nonalcoholic steatohepatitis [57,61]. In addition, it has been observed that IL-6 has the capacity to determine advanced stages of fibrosis [62]. Contrary to this, Haukeland et al. [63] discovered insignificant differences between the serum levels of the group with simple steatosis and the group with NASH. Coulon et al. [64], likewise, showed a weak correlation with NASH. However, both studies and one other study [65] revealed promising results regarding NAFLD. Concerning the role of other cytokines involved in the inflammation process, there is evidence for elevated **IL-8** serum levels in NASH too [66,67], and according to Darmadi, **IL-12** is also associated with the NAFLD ultrasound grading [68].

The cascade of inflammation involved in the pathogenesis of NAFLD and NASH involves a complex network, which includes, besides the proinflammatory cytokines, the involvement of chemokines. Chemokines are secreted by various types of cells, namely, the hepatic stellate cells, Kupffer cells, and portal fibroblasts. The chemokine activation in NAFLD results in the modulation of hepatocyte proliferation, activation, extracellular matrix remodeling, angiogenesis, and direct activation of stellate cells [69]. Since there are more than 40 chemokines with action on more than 20 discrete receptors and conflicting results in prior studies, using different methodologies, focusing on the relationship between these complex molecules and NAFLD [70], it is difficult to draw a conclusion about their current role as biomarkers of NAFLD. A systematic review and network meta-analysis conducted by Pan X et al. concluded that increased concentrations of several chemokines such as CCL2, CCL4, CCL20, CXCL8, and CXCL10 could be associated with the presence of NAFLD [71], although further research is required to establish the potential place of chemokines in the diagnostic biomarker panel for NAFLD and NASH.

Some research interest has been driven toward the study of plasma pentraxin 3 (PTX3), which represents an acute-phase protein belonging to the PTX family, significantly associated with obesity, metabolic syndrome, and cardiovascular diseases [72,73]. There have been some promising results, revealing the discriminatory power of PTX3 in detecting stage 3–4 NAFLD, with AUROC values of 0.850 [74], and also its ability to evaluate the degree of liver fibrosis [75], with a further study showing the correlation between the values of PTX3 and NAFLD activity score and fibrosis stage [76]. However, conflicting data have been reported, with a study performed by Maleki et al. [77] reporting results with no statistical significant value of PTX3 in differentiating the degree of fibrosis and with more modest results on the value of PTX3 as a sole biomarker of NAFLD (AUC-0.731); this suggests that it is better used in combination with other biomarkers in order to improve diagnostic accuracy [78].

The markers of inflammation with potential use as biomarkers in NAFLD include other molecules involved in the complex inflammatory signaling, as is the case with the **vascular cell adhesion molecule 1 (VCAM-1)**. VCAM-1 is a surface protein that induces adherence and extravasation of monocytes to blood vessels [79]. Vascular endothelial dysfunction is a significant pathophysiological component of NASH pathogenesis, with evidence from preclinical studies that endothelial inflammation is an early feature of NASH, which precedes hepatic macrophage cell infiltration [80]. There is evidence of the up-regulation of VCAM-1 expression in chronic inflammation both on the endothelial cell surface and in other types of cells, such as macrophages, dendritic cells, and Kupffer cells in the liver [81]. More recent evidence supports the hypothesis that VCAM-1 mediates the transition from steatosis to endothelial dysfunction and inflammation by facilitating the monocytes’ adhesion to the liver’s sinusoidal endothelial cells [82]. Because VCAM-1 production is not confined to the liver sinusoidal endothelial cells, this protein may affect the systemic inflammation that characterizes NAFLD [79]. Based on preclinical evidence, there is an increasing interest in integrating VCAM-1 into the biomarker panel for discriminating the progress of NASH. An analytical and clinical validation performed by Kar et al. highlighted that VCAM-1 levels were statistically significantly increased in an advanced fibrosis cohort compared with the mild and no fibrosis groups, demonstrating good clinical performance as a biomarker of advanced fibrosis and outperforming IL-6, CRP, TNFα, and chemokines [62].

Except for proinflammatory cytokines, there is evidence of the potential value of several noninvasive markers involved in various processes, such as coagulation-related markers. Plasminogen activator inhibitor-1 (PAI-1) belongs to the family of serine protease inhibitors (serpins) and is an important regulator of the plasminogen/plasmin system. The connection between PAI-1 and various diseases, including cardiovascular disease metabolic alterations, inflammation, fibrosis, and neurodegenerative disease, has been described [83]. Considering its potential role in inflammation, in a study conducted by Ajmera et al. [66], activated PAI-1 seemed to be the only marker significantly associated with the diagnosis of NASH [17].

### 3.3. Apoptosis Markers

The progression to NASH and fibrosis involves apoptotic cellular death. The extrinsic pathway is mediated by death receptors, and the interaction between the plasma membrane receptor and Fas ligand (FasL) activates the caspase cascades from which **cytokeratin 18 (CK18**), an intermediate filament protein within the hepatocytes, is cleaved in fragments of approximately 30 kDa and 45 kDa, respectively [84,85]. Therefore, CK18 could be a promising noninvasive biomarker for NASH because of the correlation between its serum level and the degree of liver damage [86]. CK18 fragments can be investigated in two ways: using immunostaining in liver tissue, or using monoclonal antibodies in plasma [87]. M30 is the specific antibody for the 30 kDa fragment and reflects cell death through apoptosis. M30 has a sensitivity of 63.6% and a specificity of 87.2% (AUC = 0.710) for predicting histological NASH [88]. High levels of CK fragments were identified in patients with NASH (AUROC = 0.93) [89], and it was suggested that it can differentiate NASH from simple steatosis [90]. It has been demonstrated that this marker is an independent predictor for NASH [91], and it is also correlated with the severity of the process [92,93]. A meta-analysis conducted by Kwok et al. reported a sensitivity and specificity of 85% and 92%, respectively, for diagnosing the F4 stage of disease using M30 [94].

Relevant data were also obtained regarding the ability of CK18 to evaluate the response to treatment. The improvement in the liver in terms of the histological aspect was correlated with the level of CK18 in two trials [13,95]. Nevertheless, Cussi et al. [96] suggested that this biomarker is not yet suitable as a screening test, since the reported results revealed lower values for sensitivity (58%) and NPV (49%) [97]. Unsatisfactory results were obtained afterward as well [98]. In order to increase the accuracy of the diagnosis of NASH, Chuah et al. [99] combined CK-18, aspartate aminotransferase (AST), and a homeostasis model assessment (HOMA), which led to the MACK-3 panel with better results. Another study reported an AUROC of 0.88 (in comparison with CK18 alone with an AUROC of 0.74) by combining metabolic syndrome, ALT, and CK18 [100]. Grigorescu et al. [101] evaluated the triple combination of adiponectin, CK18, and IL-6 and obtained an even higher AUROC (0.90).

The **fibroblast growth factor 21 (FGF21)** represents a type of endocrine fibroblast growing factor, which due to its particular structure, is able to escape the cellular matrix and enter the circulation to act as hormonal-like messengers [102]. FGF21 has been used in combination with CK18 to improve the screening process of patients with NASH [103]. Moreover, in patients with biopsy-proven NASH, a higher serum level of soluble Fas (sFas) and sFasL has been discovered compared with patients with simple steatosis [104], and this is the reason why measuring soluble Fas together with CK18 fragments increases the sensitivity (88%) and specificity (89%) in diagnostic processes [85,105]. A poor inter-test reliability represents another problematic issue for using this biomarker as a screening test at this moment, as suggested by Pimentel et al. [106]; therefore, it is not currently validated for use as a specific biomarker.

### 3.4. Adipokines and Hormones

As previously mentioned, insulin resistance plays an important role in the pathogenesis of NAFLD [107]. **Adiponectin** is an adipocytokine that regulates glucose levels and fatty acid breakdown, and whose serum level is reduced in insulin-resistant states. A meta-analysis conducted by Polyzos et al. [108] showed lower levels of adiponectin in patients with NASH compared with the control group and even with NAFLD. It has been proven that in subjects with a high likelihood of a fatty liver, adiponectin values were 40% lower than in subjects with a low likelihood of a fatty liver [109]. The trend was also observed in other studies [110,111]. Plasma adiponectin levels were associated with the degree of hepatic steatosis and necroinflammation but not with the severity of fibrosis [112]. Adiponectin was, again, negatively correlated with the stage of NASH in obese patients [113]. Nevertheless, hypoadiponectinemia in NAFLD has been proven to be the consequence of metabolic disturbances and fat accumulation in the liver more than just the severity of the disease [114,115]. A combined evaluation (serum adiponectin level, HOMA-IR, and serum type IV collagen 7S) seemed to obtain even better results in terms of sensitivity (94% vs. the sensitivity of adiponectin alone, 68%) [116]. As we mentioned in the “CK-18” section, adiponectin may be also integrated into a panel alongside CK-18 and IL-6 [101].

Among key players involved in insulin resistance, the value of the **adipocyte fatty acid-binding protein (AFBP)** was assessed in the serum of NAFLD patients, and elevated values were found in this patient category. Moreover, evidence suggests that AFBP could predict fibrosis [117]. The same study reveals that **lipocalin-2**, another tissue-derived, lipid-binding cytokine, can differentiate NAFLD from the control group as well, but it is not able to distinguish NASH from simple steatosis. Ye et al. also found a correlation between NAFLD and lipocalin-2 [118].

**Visfatin** is a multifunctional adipocytokine that is produced and secreted in visceral fat, and it is correlated with metabolic syndrome [119]. The study conducted by Jarrar et al. [111] suggests that visfatin and IL-6 may be co-regulated and identified lower values of visfatin in NASH compared with simple steatosis. According to Aller et al. [120], visfatin plasma concentrations could predict the presence of portal inflammation in NAFLD patients. **FGF-21** is increased in obese patients, and it is correlated with the hepatic fat content and with the nonalcoholic fatty liver disease activity score [121]. As mentioned beforehand, FGF21 could represent a helpful biomarker for NAFLD, with a reported AUROC of 0.84 for detecting NAFLD [103] and with higher diagnostic accuracy when combined with CK-18. A meta-analysis suggests its ability to detect NASH; however, the number included was low, which weakens the strength of the conclusion [122]. **Leptin** represents an adipocytokine involved in lipid accumulation and inflammation, and it has been observed that it is a predictor for the severity of hepatic steatosis but not of hepatic fibrosis [123]. Another study was, however, inconclusive [124]. Regarding **resistin**, there are conflicting results. According to Argentou et al., it is negatively correlated with the NAFLD activity score and steatosis grade but not with inflammation [113], as Aller et al. [125] also claim. Nevertheless, a positive correlation was found between resistin and the histological inflammatory score [126]. **Dehydroepiandrosterone (DHEA)** is a steroid hormone that among other functions has been reported to augment insulin sensitivity. It seems that DHEA could suggest the progression of NAFLD into NASH and advanced fibrosis, considering the results of a study revealing lower levels of DHEA-s (the sulfated form) in more histologically advanced NAFLD. The results were correlated with age, as it is known that DHEA decreases with time [127]. Other significant results were obtained regarding the **insulin-like growth factor-binding protein 1 (IGFBP-1)**, as it has been observed that IGFBP-1 was associated with the presence of advanced fibrosis, which makes it a promising future biomarker [128].

### 3.5. Biomarkers of Lipid Oxidation

It has been established that the pathogenesis of NAFLD involves oxidative stress characterized by an imbalance between prooxidants and antioxidants, leading to hepatic injury and underlying the development of NASH. We can therefore potentially evaluate the pathways and quantify the metabolites involved in this process in order to assess the liver status [129]. This statement is supported by several clinical studies, where various biomarkers of lipid oxidation have been studied. Several studies focusing on this topic have included the evaluation of various markers, such as the total antioxidant response and total peroxide level [130] and the measurement of **9- and 13- Hydroxyoctadecadienoic acids (9- and 13-HODEs)** and **9- and 13-oxoODEs**, which are products of free-radical-mediated oxidation of linoleic acid (LA) [131]. Their results suggest that although no correlation was observed between the necroinflammatory grade and these oxidative status parameters’ elevated values [130], the values of **9- and 13-HODEs** and **9- and 13-oxoODEs** have been increased in patients with NASH [131], suggesting the potential use of these parameters in diagnosing NASH. In the quest to capitalize on the role of oxidative stress in NAFLD progression, clinical studies identified that the serum level of **malondialdehyde (MDA)** [132] is increased in NAFLD, as this highlights that lipid peroxidation represents a marked process and, more than that, a decrease in the levels of antioxidants such as Coenzyme Q10 (**CoQ10)** and **CuZn-superoxide dismutase (SOD)** [133]. Regarding antioxidant enzymes, **erythrocyte GSH**, **SOD**, and **catalase** seemed to be significantly lower in biopsy-proven NAFLD [134], as did the serum paraoxonase 1 activity [132], with some results suggesting that MDA and GSH potentially represent independent risk factors for fibrosis in NASH [135]. Nevertheless, this could also be the case when only the oxidation processes are increased in order to produce an imbalance [136]. Direct measurements of **oxidized LDL (ox-LDL)** and **thiobarbituric acid-reacting substances (TBARSs)** revealed elevated values in NASH [137]. Alkhouri et al developed a diagnostic score called **Ox-NASH**, reporting its potential to predict the presence of NASH and also correlate with NASH histology [138]. This score is calculated from the ratio of 13-HODE to linoleic acid, age, BMI, and AST. Videla et al. emphasized the presence of oxidative stress in NAFLD and especially in NASH by providing relevant data: **reduced glutathione (GSH) content**, **superoxide dismutase (SOD) activity**, and the **ferric-reducing ability of plasma (FRAP)** were decreased in this study [139]. In the same study, a potential role of the induction of **cytochrome P450 2E1 (CYP2EI)** in the context of this condition was suggested because of the significantly high values of these parameters compared with controls. Chtioui et al. [140] also confirmed this finding by using a 6-hydroxychlorzoxazone/chlorzoxazone (CHZ) ratio (CHZ test), but they highlight the fact that it is unable to differentiate steatohepatitis from simple steatosis. At the same time, Orellana et al. [141] evaluated both liver Cytochrome P450 2E1 (CYP2E1) (through Western blot) and CHZ hydroxylation in obese patients with steatosis, and they also found an enhanced activity. Albano et al. [142] used the immune response to assess the lipid peroxidation. They utilized circulating IgG against lipid peroxidation products such as titers **of IgG against human serum albumin adducted with malondialdehyde (MDA-HSA)** or **arachidonic acid hydroperoxide (AAHP)** and **against oxidized cardiolipin (Ox-CL)**, and the levels seemed to be significantly higher in NAFLD patients. An interesting finding was that the titers of these markers were independent of the steatosis grade but in accordance with fibrosis.

Looking at oxidative stress from a different angle and targeting noninvasiveness in the investigation, research on the role of oxidative stress in NAFLD has also included the evaluation of **volatile organic compounds (VOCs)**, which are considered markers of oxidative stress. Analyzing VOCs in exhaled breath seemed useful for indicating the presence of NASH, with one study examining several VOCs: n-tridecane, 3-methyl-butanonitrile, and 1-propanol. The obtained results were promising, with an AUROC of 0.77, a negative predictive value of 82%, and a positive predictive value of 81% in discriminating NASH [143].

The role of all these biomarkers reflecting lipid oxidation in NAFLD is still to be integrated into the biomarker panel, and their use in discriminating NAFLD stages is still to be evaluated, with lipidomics appearing to be a promising lane in this direction.

### 3.6. Genetic Biomarkers

The fact that the prevalence and severity of NAFLD are variable among individuals and ethnic groups (there are higher rates in Hispanic populations compared with Caucasians) points to the role of genetics in the pathophysiology of NAFLD [144], with a twin study also implying the heritability of NAFLD [145]. There have been a significant number of genome studies that provided great data on new potential biomarkers [90]. Both Younossi et al. and Sreekumar et al. have shown that there are genes with a significantly differential expression in NASH [146,147]. Bragoszewski et al. conducted a study regarding the expression of genes encoding mitochondrial proteins. They concluded that the **expression of HK1, UCP2, ME2, and ME3** appeared to be higher in NASH than in NAFLD patients, whereas **HMGCS2 and hnRNPK expression** were contrarily lower [148]. Greco et al. [149] have also studied gene expression in NAFLD and discovered 1060 genes related to carbohydrate, lipid, or amino acid metabolism that are significantly associated with the liver’s fat content. An increased expression of the genes involved in inflammation and mitochondrial alterations was, again, highlighted by Chiappini et al. [150] in a study that used high-density oligonucleotide microarray. The same method was used by Yoneda et al. as well [151]. Not only are the genes involved in lipid metabolism and inflammation over-expressed in NAFLD and NASH, the expression of the genes involved in monocyte/macrophage recruitment is also increased, as Westerbacka et al. suggest [152].

In addition, the DNA copy number and deletion levels also appear to be associated with the NAFLD settlement. The number of copies was significantly higher in NAFLD patients compared with healthy controls, and 4977-bp deletion has been discovered in 8 out of 43 patients with the condition, but meanwhile in none of the control groups [153]. A Chinese study found that the copy number of mitochondrial DNA is associated with the incidence of NAFLD only under the influence of 8-oxo-2′-deoxyguanosine (8-oxo-dG), highlighting the impact of oxidative stress in the pathogenesis of NAFLD [154]. The mitochondrial genome is transcripted in the D-loop region, which has been explored by Hasturk et al., who identified the m.A16318C variant only in patients with NASH [155].

The most common studies imply genetic variations in the form of single nucleotide polymorphisms (SNPs). These modifications present at birth and could be used in large screening programs [10]. The **PNPLA3 gene** encodes Patatin-like phospholipase-domain-containing protein 3 (PNPLA3), also known as adiponutrin, that may be involved in the balance of energy usage/storage in adipocytes. **Rs738409 (an I148M protein variant)** represents the isoleucine-to-methionine substitution in the PNPLA3 gene at position 148, which induces a loss of function in the enzymatic activity and as a consequence, the abnormal storage of triglycerides [156,157]. It is most prevalent in Hispanic populations (49%) [158] and is associated with a systematic increase in hepatic fat content but without a connection with lipoprotein metabolism [159]. The carriers of this variant are at high risk of developing steatohepatitis, cirrhosis, and HCC [160,161]. A meta-analysis conducted by Xu et al. confirms the susceptibility of PNPLA3 rs738409 polymorphism to NAFLD and the risk of developing NASH [162], and the results are consistent with a previous meta-analysis [163]. Nevertheless, there is a positive aspect related to the presence of this variant: its carriers are advantaged by the fact that weight loss is more effective in decreasing liver fat in subjects who are homozygous for the rs738409 PNPLA3 G or C allele [164,165]. The NASH Clin Score represents a model of diagnosis that associates the PNPLA3 G genotype with clinical features such as aspartate AST level and fasting insulin, with promising values such as a reported AUROC for detecting NASH of 0.792 [166].

The transmembrane 6 superfamily member 2 (TM6SF2) is located on chromosome 19 (19p12); one variant of TM6SF2 (E167K, rs58542926), which is carried by about 10% of individuals [167], has been shown by genetic studies to be associated with the hepatic triglyceride content [168]. A point mutation in TM6SF2 (rs58542926, c.499 C > T) causes glutamine-to-lysine substitution, which has as a translational result a misfolded protein, leading to accelerated protein degradation [169]. Studies reveal that this variant has a negative effect on lipids metabolism, contributing to the reduction of the secretion of very low-density lipoproteins (VLDLs) responsible for the accumulation of triglycerides in the liver. Therefore, it further induces the development of NAFLD and eventually NASH or fibrosis [169,170,171,172,173,174]. However, this results in a decreased level of lipids in the circulation, and therefore, a reduced risk of developing myocardial infarction [175].

**Rs780094 A > G** and **rs1260326 C > T variant (P446L)** occur in the **glucokinase regulator (GCKR) gene**, which is involved in the regulation of the glucose influx to hepatocytes. The effect of this variant consists of an inability to regulate the glucose influx into hepatocytes, resulting in an accelerated uptake and an increase in the production of malonyl-CoA and de novo lipogenesis, with the consequence of promoting the development of NAFLD, NASH, and fibrosis [144,176,177]. Stender et al. [178] suggest that the effects of all of these three sequence variants (PNPLA3 p.I148M, TM6SF2 p.E167K, and GCKR p.P446L) are amplified by the presence of adiposity, which further promotes the deterioration towards NASH and cirrhosis.

The **rs641738 C > T** variant of the **membrane-bound O-Acyltransferase domain containing 7 (MBOAT7)**, which is involved in phospholipid metabolism, is also associated with an increased risk of the entire spectrum of NAFLD [179,180], including higher chances of developing HCC in patients without advanced fibrosis [181]. Additionally, the gene encoding the enzyme hydroxysteroid 17β-dehydrogenase 13 (HSD17B13), which is involved in steroid and lipid metabolism, is overexpressed in NAFLD and causes lipid accumulation in the liver. Interestingly, it seems that a loss-of-function variant could have a protective effect regarding the progression to steatohepatitis [2].

It is important to know and understand the role of genetic polymorphisms in NAFLD; current relevant findings are presented in Table 2.

These findings can be integrated into algorithms for the diagnosis of the NAFLD spectrum. One example is FibroGENE, which is a gene-based model capable of staging liver fibrosis and which obtained satisfactory AUROCs compared with APRI or FIB-4 [183]. The risk of developing a hepatocellular carcinoma from NAFLD can be assessed using the Polygenic Risk Score-5, which combines PNPLA3, TM6SF2, GCKR, and MBOAT7 [184].

### 3.7. Role of Epigenetics

Besides genomics, it has been shown that epigenetics plays a meaningful role in NAFLD pathophysiology. Epigenetics refers to the diversity of gene expression not related to the DNA sequence itself and includes **DNA methylation** differences, which could be the reason for overexpression or underexpression of genes [185], **histone modifications**, and **noncoding RNAs** [17,186]. For NAFLD, several studies have evaluated whether the differential DNA methylation at the peroxisome proliferator-activated receptor Gamma (PPARγ) promoter can be detected within the pool of cell-free DNA of human plasma to avoid taking a biopsy. Hardy et al. [187] answered positively to that question and brought to light the possibility of DNA methylation of PPARγ to noninvasively stratify liver fibrosis. The inclusion of the DNA methylation to contribute to staging fibrosis was also confirmed by Johnson et al. [188] in a study that showed a sensitivity of 0.93 for predicting fibrosis. The nuclear genome does not represent the only source for DNA methylation, since the methylation process can also affect the mitochondrial DNA. Mitochondrial dysfunction seems to be involved in the progression of NAFLD, and according to Pirola et al. [189], NADH dehydrogenase 6 (MT-ND6) encoded in mitochondria is more methylated in the liver of NASH patients compared with those with simple steatosis. This impacts the transcriptional regulation of mitochondrially encoded NADH dehydrogenase 6 (MT-ND6), and therefore, it has been shown to be correlated with the NAFLD activity score [189]. Ahrens et al. [190] observed differences in the methylation of pyruvate carboxylase, ATP Citrate Lyase (ACLY), and phospholipase C Gamma 1 (PLCG1) genes and also noticed the impact of bariatric surgery on inducing changes in their methylation status. The peroxisome proliferator-activated receptor C coactivator 1a (PPARGC1A) and the mitochondrial transcription factor A (TFAM) promoters were the subjects of investigation for Sookoian et al. [191]. They showed that the hepatic methylation of these two genes is associated with insulin resistance, which is a well-known player in the development of NAFLD. The steatosis degree of the liver can also be assessed via epigenetic changes; Ma et al. [192] showed a correlation between hepatic fat and the methylation status of 22 CpG. Furthermore, the hypomethylation at Acyl-CoA synthetase long-chain family member 4 ACSL4 (cg15536552) is suggested to be a potential biomarker for NAFLD progression [193]. Hyun et al. reviewed the gene methylation signatures in NAFLD, underlining their potential as therapeutic targets as well as biomarkers useful in staging NAFLD, while needing a better understanding of their place in NAFLD pathogenesis [194].

Another promising biomarker is **circulating cell-free DNA**, which proved useful in assessing the disease severity in a study including patients with NAFLD diagnosed with standard noninvasive methods [195]. Acetylation, modulated by **histone acetyltransferases (HATs)**, represents the main reaction suffered by histones whose result promotes transcription. In conditions of hyperglycemia, the transcriptional coactivator p300 acetylates the carbohydrate-responsive element-binding protein (ChREBP) and implicitly increases lipogenic activity, which eventually leads to NAFLD installation [17,196]. On the other hand, **histone deacetylases (HDACs)** include **sirtuins**, which protect against NAFLD. Nevertheless, a deficiency of SIRT3, which is a type of sirtuin, has been shown to lead to insulin resistance and steatohepatitis, and SIRT3 polymorphisms seemed to lead to NAFLD [196].

**Noncoding RNAs** include **microRNAs (miRNAs)** and **long noncoding RNAs (lncRNAs)** and interfere with transcriptional activity [196]. Apparently, the severity of NAFLD is, among other factors, a consequence of the disturbances in microRNA expression [197,198,199]. **Transcriptomics** is relevant for noninvasively diagnosing NAFLD, mainly through miRNAs, as it can be reflected in the peripheral blood [196], since it is very stable and, in addition, their measurement is performed through a very sensitive method [200]. Some studies illustrated the correlation between serum miR-122, -192, and -34a and steatosis or inflammatory activity [201,202]. The latter obtained an AUROC of 0.811 [202] for diagnosing NASH, a superior value compared with that of CK-18 and also a 7.2-fold change in NASH vs. controls [203], suggesting its promising potential as a noninvasive biomarker [204]. These results consolidated the final conclusion of Yamada et al., who identified that miR-122 has a good capacity for staging liver steatosis [205].

Elevated levels of mi-RNA-122 and mi-RNA-34a were obtained among NAFLD patients, along with satisfactory results for the sensitivity and specificity (92% and 85%, respectively) of mi-RNA-122, as opposed to mi-RNA-99a levels, which were found to be decreased and could be used as a tool to discriminate simple steatosis from steatohepatitis [206]. Furthermore, miRNAs have been included in a score (NIS4TM) that covers parameters involved in different processes included in the pathogenesis of NAFLD, such as inflammation, hepatocyte ballooning, or tissue remodeling. This score includes the evaluation of miR-34a, together with alpha-2 macroglobulin, YKL40, and HbA1C [207]. Serum miRNA-122 level has been proven to be correlated with the histopathologic features of NAFLD; an improvement in the liver biopsy aspect seemed to be associated with lower values of the circulating miRNA-122 [208]. A meta-analysis conducted by Liu et al. [209] revealed consistency in results for miRNA-122 and miRNA-192 in discriminating NAFLD from NASH. Additionally, miRNA-122 can be contained in extracellular vesicles, which makes it an even more reliable biomarker [210]. miRNA-21 interferes with lipids metabolism through the inhibition of a key enzyme in the cholesterol synthesis process, namely, the expression of 3-hydroxy-3-methylglutaryl-co-enzyme A reductase [211]. A study performed in the Chinese population highlighted a correlation between the serum level of miRNA-29b and the liver fat content [212]. miRNA-132 represents another evaluated type of mRNA, which has shown the same potential in the screening and diagnosis of NAFLD [213]. A selection of relevant miRNAs useful in the diagnosis of NAFLD can be found in Table 3.

In order to acquire a more precise ability to predict and discriminate NASH, miRNA expression profiles were combined, with a concomitant evaluation of various types of miRNA, with the resulting panel obtaining an AUC of 0.856 [197], or with other standard markers such as CK18 obtaining a positive outcome in this regard [220].

As far as **lncRNA** is concerned, Sun et al. conducted a clinical study using microarray expression profiling to evaluate lncRNA, highlighting different expression profiles in patients with NAFLD compared with patients without this condition [221]. They also validated a few of these differentially expressed lncRNAs via qRTPCR, and the results were consistent. lnc18q22.2 appears to be a liver-specific lncRNA with an increased expression in the hepatocytes of NASH patients, correlating with the NASH grade and lobular inflammation and providing new insights into the regulation of hepatocyte viability in NASH [222]. Considering all this evidence, circRNAs (circular RNAs) represent a new field of research in the matter of noninvasive biomarkers for NAFLD and NASH, but further studies are needed [223].

### 3.8. Omics in NAFLD

#### 3.8.1. Proteomics

Since the gene expression does not entirely reflect the totality of the proteins in the cell, the newly developed proteomic technologies are more representative of the phenotype [196]. There are promising data about proteomics which make this field valuable for discovering new biomarkers for NAFLD [224]. A study including obese patients revealed three protein peaks (CM10-7558.4, CM10-7924.2, and Q10-7926.9) with elevations proportional to the progression of liver disease. A normalization of the values of these protein profiles was also identified after bariatric surgery. These peaks have been proven to be characterized as double-charged ions of a- and b-hemoglobin subunits [225]. Bell et al. used an ion-intensity-based, label-free quantitative proteomics approach (LFQP) to analyze 605 proteins, which were expressed differently between any two groups involved, emphasizing 15 proteins that discriminate between patients with NASH and NASH with advanced fibrosis [226]. The functions of these proteins consolidate our knowledge about the various and complex pathways of NAFLD’s pathogenesis, and so do the diverse proteins identified in the plasma proteome profiling conducted but Niu et al. [227]. A study including the analysis of the hepatic expression of significant proteins revealed that lumican, a keratin sulfate proteoglycan, is progressively overexpressed among NAFLD stages. In order to assess the liver specificity of lumican, IHC staining was used in this study [228]. Wood et al. combined genomic, phenomic, and proteomic data and demonstrated that a multi-approach diagnostic has a better predictive ability [229]. With the help of the SOMAscan proteomics platform, 1305 proteins with different expressions have been discovered and have been further used to classify fibrosis stages [230].

#### 3.8.2. Glycomics

The proteins suffer post-translational changes, and glycosylation represents an important reaction that instates the variability of structures and functions. Since many glycoproteins are formed in the liver, the alterations of glycosylation might indicate liver dysfunction [231]. The **serum N-glycan profile** is a valuable tool for identifying NASH, since there are data regarding different concentrations of some glycans among patients with SS vs. NASH. Based on glycomics, a fibrosis score called **GlycoNashTest** has been developed [232].

#### 3.8.3. Lipidomics

Due to the fact that the pathogenesis of NAFLD implies the accumulation of triglycerides, lipidomics represents an important field of metabolomics used for searching for new noninvasive methods of diagnosis [17], especially as NAFLD seems to have a characteristic lipidomic profile. It has been shown that the **enzyme desaturase FADS1** exhibits decreased activity, which is responsible for the accumulation of fatty acids in the liver and for the disturbances in phospholipid synthesis [233]. With the intention of evaluating the plasma lipidome of NAFLD, Puri et al. identified an elevated level of total plasma monounsaturated fatty acids [234]. Mayo et al. developed a lipidomic test, consisting of 28 TGs, which showed an AUROC of 0.95 for discriminating between NAFLD and NASH [235]. A previous large study suggested that **saturated TG (16:0/18:0/18:1)** would be the best predictor among the TGs. Its combination with phosphatidylcholine [PC] [18:1/22:6] and PC [O-24:1/20:4] constitutes a lipid triplet model with a satisfactory predictive value [236]. **Plasma eicosanoids** also seem to be useful for assessing liver fibrosis [237]. Since hepatocellular ballooning represents such an important histological process, NAFLD patients can be classified by the presence or absence of this finding, as this aspect is relevant for the disease progression, prognosis, and treatment. Ogawa et al. assessed the hepatocellular ballooning using the increased plasma level of phosphatidylcholine (PC) (aa-44:8) found in NAFLD and the lower plasma level of lysophosphatidylethanolamine (LPE) (e-18:2) [238]. Yamada et al. [239] discovered that the **serum C16:1n7/C16:0 ratio** in NASH patients is statistically correlated with liver histology and has an AUROC for predicting NASH among NAFLD patients of 0.709.

It is a well-known fact that obesity has been linked with NAFLD prevalence and severity [240], with one study highlighting that **isolated saturated sphingomyelin (SM)** species are biomarkers for predicting NASH in the nonobese patient group, showing the specificity of lipidomic profiles according to obesity status [241].

Promising results were obtained for pyroglutamate in a study in which 55 other metabolites were discovered to have the ability to discriminate between NASH and simple steatosis. The capacity of **pyroglutamate** for predicting NASH seemed to compete with that of several other markers, such as TNF-α, adiponectin, and IL-8. The obtained accuracy was 82%, with sensitivity and specificity of 72% and 85%, respectively [58].

#### 3.8.4. Metabolomics

The association of NAFLD with metabolic syndrome has been extensively studied, and allegedly, NAFLD might be the liver expression of metabolic disturbances [242]. This fact represents an important diagnostic aspect. Through a metabolomic analysis, an elevated plasma level for **glycocholate, taurocholate, and glycochenodeoxycholate** as well as a lower value for glutathione were identified in NASH patients [243]. Competing with the FIB-4-index and NFS, a 10-metabolite panel consisting of 8 lipids (5alpha-androstan-3beta monosulfate, pregnanediol3-glucuronide, androsterone sulfate, epiandrosterone sulfate, palmitoleate, dehydroisoandrosterone sulfate, 5alpha-androstan-3beta disulfate, glycocholate), 1 amino acid (taurine), and 1 carbohydrate (fucose) showed a great potential in noninvasively assessing advanced fibrosis, which consolidates the importance of metabolomics in the future identification of NAFLD’s serum biomarkers [244]. Associating the evaluation of glutamate, isoleucine, glycine, 20 lysophosphatidylcholine 16:0, and phosphoethanolamine 40:6 with the already discussed NASH Clin Score, a more accurate predictive model called NASH ClinLipMet is obtained. This complex evaluation evaluates the NASH patients more accurately, due to the balanced combination of clinical, genetic, and metabolic features [166].

## 4. Conclusions

There are a plethora of biomarkers with evidence indicating a potential to contribute to evaluating disease severity in NAFLD. Many of these biomarkers are of use in other pathological entities as well, since common pathophysiological mechanisms are involved. Therefore, the mentioned biomarkers have little discriminatory power when used individually. Therefore, a combination of biomarkers reflecting several mechanisms involved is required. This combined approach, with the inclusion of several biomarkers in scores, contributes to increasing diagnostic accuracy.

The early identification of steatohepatitis and fibrosis is a key step in the management of NAFLD in order to prevent disease progression. From this point of view, considering the prognostic significance of steatohepatitis and fibrosis, it is mandatory to identify the optimum combination of biomarkers that can suggest the presence of steatohepatitis and quantify the fibrosis in the least invasive manner.

Subsequent work should identify algorithms to integrate several biomarkers involved in various branches of the complex pathophysiology of NAFLD and NASH in order to comprehensively evaluate the disease severity in a noninvasive manner and offer a predictive value for disease evolution.

## Figures and Tables

**Table 1:** Currently used scores based on serum biomarkers to evaluate NAFLD, NASH, and liver fibrosis.

Parameters Involved	FibroTest [16]	FibroMeter [10]	Hepascore [16]	NAFDL Fibrosis Score [16]	BARD Score [16]	AST/Platelet Ratio Index (APRI) * [17]	Hepamet Fibrosis Score [18]
Liver function tests	Bilirubin GGT	Serum levels of ALT, AST	GGT Bilirubin	AST/ALT ratio Serum albumin level	AST/ALT ratio	AST elevation	AST (UI/L) Albumin (g/dL)
Anthropometric data	Age	Body weight	Age Sex	Age BMI	BMI		Age Sex
Parameters of metabolic comorbidity		Fasting glucose		Diabetes mellitus status Fasting glucose level	Diabetes mellitus status		Insulin (μU/mL) HOMA Diabetes mellitus status Glucose (mg/dL)
Other parameters	Apolipoprotein A1 α2-macroglobulin	Ferritin Prothrombin index	HA a2-macroglobulin	PLT count		PLT count	PLT (×10^9^)

* (AST elevation/PLT count) × 100. ALT—alanine aminotransferase; AST—aspartate aminotransferase; BMI—body-mass index; GGT—γ-glutamyl transpeptidase; HA—hyaluronic acid; PLT—platelet; HOMA—Homeostatic Model Assessment for Insulin Resistance.

**Table 2:** Genes and their altered variants involved in NAFLD pathogenesis.

Gene	Role of Encoded Protein(s)	Variant	Effect of Altered Gene Variant
PNPLA3 [182]	Patatin-like phospholipase-domain-containing protein 3 (PNPLA3) = adiponutrin → involved in TG and retinoid metabolism	rs738409	Abnormal storage of triglycerides
Transmembrane 6 superfamily member 2 (TM6SF2) [182]	TM6SF2 protein → involved in secretion of apolipoprotein B	rs58542926 C > T→ a loss-of-function mutation	Higher liver TG content and lower circulating lipoproteins
Glucokinase regulator (GCKR) gene [182]	Glucokinase regulatory protein (GKRP) → involved in the regulation of glucose influx to hepatocytes (2)	rs780094 A > G and rs1260326 C > T variant (P446L)	Increased hepatic glucose uptake and increased de novo lipogenesis
Membrane-bound O-Acyltransferase domain containing 7 (MBOAT7) [182]	Lysophosphatidylinositol (LPI) acyltransferase 1 → involved in the regulation of free arachidonic acid	rs641738 C > T→ a loss-of-function mutation	Increased free polyunsaturated fatty acids and proinflammatory eicosanoid lipids
HSD17B13 [182]	A lipid droplet enzyme retinol dehydrogenase	rs72613567:TA	Decreased inflammation, Ballooning, and fibrosis → a protective variant
Apolipoprotein B (APOB) [144]	ApoB protein → involved in the formation and secretion of hepatic VLDL and chylomicrons	Loss-of-function mutations	Decreased serum cholesterol and increased intrahepatic triglycerides
Proprotein convertase subtilisin kexin 9 (PCSK9) [144]	Serine protease → promotes the degradation of LDL receptors	Loss-of-function mutations	Decreased serum cholesterol
Microsomal triglyceride-transfer protein (MTTP) [144]	A lipid transfer protein → involved in VLDL particle formation; chaperone for apoB	Different mutations	Abetalipoproteinemia and accumulation of hepatic triglycerides
APOC3 [144]	Apolipoprotein C3 (apoC3) → involved VLDL particles formation	Different mutations	Hypertriglyceridemia
LIPA [144]	Lysosomal acid lipase (LIPA) → hydrolysis cholesteryl esters, triglycerides, and LDL particles	Loss-of-function mutations	Hypercholesterolemia, cardiovascular disease, hepatic steatosis, and cirrhosis
FATP5 [144]	Fatty acid transport protein → increases hepatic fatty acid uptake	rs56225452	Promotes hepatic steatosis and determines insulin resistance
LPIN1 [144]	A phosphatidate phosphatase transcriptional coactivator, which controls fatty acid metabolism	rs12412852	Reduced lipolysis

**Table 3:** miRNAs with diagnostic value for NAFLD.

miRNA	Expression	Role	Predictive Value
miR-122 [214]	Up-regulated	Regulates several genes involved in the lipid metabolism such as fatty acid synthetase, acetyl-coenzyme-A carboxylase-2, or HMG CoA reductase	Indicator for steatosis and fibrosis stage
miR-34a [214,215]	Up-regulated	Down-regulates the PPARα signaling pathway and induces lipid accumulation in hepatocytes	Indicator for fibrosis, steatosis, and inflammation
miR-16 [216]	Up-regulated in NASH but down-regulated in fibrosis	Inhibits the expression of several anti-apoptotic genes	Correlated with liver inflammation and useful for predicting NAFLD-HCC progression
miR-21 [217]	Up-regulated	Regulates hepatic fat accumulation targeting HMGCR, FABP7 [218]	Indicator for fibrosis and inflammation
miR-10b [219]	Up-regulated	Regulates the nuclear receptor called peroxisome proliferator-activated receptor-*α* (PPAR-*α*)	Indicator for steatosis
miR-192 [209]	Up-regulated	A target of TGFβ1 [216]	Discriminator between NAFLD and NASH
miR-29b [212]	Up-regulated	Regulates lipid metabolism	Indicator for steatosis
miR-132 [213]	Up-regulated	A target of Sirt1	Indicator for steatosis
miR-199a [219]	Up-regulated in NASH and fibrosis but down-regulated in steatosis	A target of NCOR1 [218]	Positively correlated with fibrosis

FABP7—fatty acid-binding protein 7, HCC—hepatocellular carcinoma, HMGCR—3-hydroxy-3-methyl-glutaryl-coenzyme A reductase, PPARα—peroxisome proliferator-activated receptors, NCOR1—nuclear receptor corepressor 1, Sirt1—sirtuin-1, TGFβ1—transforming growth factor-beta.
